# Editorial: Human organoid technology for virus research

**DOI:** 10.3389/fcimb.2023.1155252

**Published:** 2023-03-08

**Authors:** Dasja Pajkrt, Veronica Krenn, Joana Rocha-Pereira

**Affiliations:** ^1^ OrganovirLabs, Pediatric Infectious Disease, Amsterdam University Medical Centers location Academic Medical Center, University of Amsterdam, Amsterdam, Netherlands; ^2^ Human Technopole Early Career Fellow, Department of Biotechnology and Bioscience, University of Milan-Bicocca, Milano, Italy; ^3^ Laboratory of Virology and Chemotherapy, Department of Microbiology, Immunology and Transplantation, Rega Institute, Katholic University Leuven, Leuven, Belgium

**Keywords:** human organoids, virus infection, gut organoid, antiviral testing, respiratory tract organoid

Traditionally, human virus research has relied on results generated from immortalized cell lines and animal models experiments. For new (antiviral) drugs to become available on the market as registered treatments for patients, these candidate drugs needed to be tested first in animals. As per late December 2022, a law signed by President Joe Biden in the United States of America stated that new drugs do not need to be tested in animals before approval by the US Food and Drug administration (FDA). This law allows the FDA to facilitate a drug to human trials after either animal or nonanimal tests (www.science.org/content/article/fda-no-longer-needs-require-animal-tests-human-drug-trials).

The approval of antiviral results in nonanimal models to enter human trials is a major step in reducing unnecessary animal testings and hopefully improving translatability of novel *in vitro* models.

One of these models that have the potential to recapitulate the complex human physiology are human organoid models.

Over the last years an increase in the application of human organoids models for studies of human viral pathogenesis and antiviral testing has been very apparent.

In this special Frontiers Research Topic, we focused in-depth on the use of human gut and respiratory tract organoid technology to study human viral infections. Here, researchers demonstrate that indeed new important conclusions can be drawn based on this new human organoid technology.

In the first article by Roodsant et al, a new human 2D gut epithelial transwell system derived from human organoids was developed for studies on host-pathogen interactions. Using human fetal intestine tissue, fetal organoids were generated, mechanically disrupted and seeded on Transwell cell culture transwells. The monolayer model represented characteristics of human intestinal epithelium, including formation of a tight epithelial barrier (as measured by Transepithelial Electrical Resistance), epithelial polarization, presence of specialized intestinal cells (goblet cells, Paneth cells, enteroendocrine cells, and stem cells), and gene expressions that characterized fetal intestinal tissue of origin. The developed organoid-derived gut epithelial transwell monolayer showed to be suitable for studying both bacterial and viral gastrointestinal pathogens, as demonstrated with the infection of the monolayers with *Listeria monocytogenes and* Enterovirus A71 (EV-A71).

In the second article from the OrganovirLabs group in Amsterdam, the Netherlands, Garcia-Rodriguez et al., aimed to increase the knowledge on the disease mechanisms of human parechoviruses (PeV-A). PeV-A1 is known to elicit gastrointestinal disease in young children, and PeV-A3 is linked to severe disease (meningo-encephalitis, sepsis-like illness) in infants and neonates. As PeV A1 and A3 are detected in human stool and nasopharyngeal swabs, it is hypothesized that the primary entry sites of PeVA are the intestine and respiratory tract respectively. The authors used the above described human 2D organoid-derived gut epithelial transwell system for host-virus studies of human parechoviruses (PeV-A) infection. The transwell model was permissive to infection PeV-A1 and clinical isolates of PeV-A3. The viral replication rate was highest after infection of the transwell system from the basolateral side as compared to the apical site. Compared to PeV-A1, PeV-A3 replicated slower. PeV-A1 infected both Paneth cells and enterocytes, while PeV-A3 infected mainly goblet cells. The authors concluded that the differences in cell tropism may influence the viral replication rates and the differences in disease as seen in humans.

In the third article, Ekanger et al. aimed to study respiratory viral infections by developing a human adult stem cell-derived organoid model representing the upper respiratory airways and lungs. This novel model was first fully characterized in the context of growth, cellular composition and functionalities. Next, several viral entry receptors such as the influenza virus-relevant sialic acids and SARS-CoV-2 entry receptor ACE2 and TMPRSS2, were illustrated to be present in this new human organoid model of bronchioli and alveoli. Infection of the pseudotype influenza A H7N1 and H5N1 virus, and the ability of the model to support viral replication of influenza A H7N1 virus was demonstrated. Finally, infection of the organoid model with SARS-CoV-2 was established. The authors conclude that this new human adult stem cell-derived organoid model may be relevant and reliable models for virus research and of added value in pandemic preparedness and antiviral testing.

In the last article, Clifton et al. used a pseudostratified mucociliated mucosal barrier model to identify differentiation stage-specific biomarkers. By applying transcriptomic analyses, the authors showed that expression pattern and magnitude of genes such as OMG, KRT14, STC1, BPIFA1, PLA2G7, TXNIP, S100A7 indicate the stage of epithelial cell differentiation. These findings were validated in multiple donors using quantitative hemi-nested real-time PCR. Finally, authors state that increases in BPIFA1 secretion correlated with the emergence of secretory cells and an anti-inflammatory phenotype, as airway epithelial cells undergo mucociliary differentiation under air-liquid interface *in vitro*.

Taken together, the four articles within this special Frontiers Research Topic show that human derived organoid technology can be successfully applied for virus research and may have the potential to translate into disease in humans.

## Author contributions

DP, VK and JR-P drafted the work or revised it critically for important intellectual content. All authors contributed to the article and approved the submitted version.

